# Medical Items and News of Interest to the Physician

**Published:** 1879-01

**Authors:** 


### For the Use of Physicians.

CULLED FROM THE STANDARD MEDICAL JOURNALS OF THE WORLD.

NOTE.—These formulæ are intended only for the regular practitioner of medicine, and should be employed only by him, or under his immediate supervision.

**Creasote-Wine.**

Beechwood-tar creasote, 192 grains.
Tincture of gentian, 1 fl. ℥.
Alcohol, 85 per cent, 8 fl. ℥.
Malaga wine, to make one quart.

**Ivy Poisoning.**

A saturated solution of chlorate of potassa, applied locally to the affected parts, is recommended by a correspondent in the *Drug. Circular.* By this means he is sure to cure or greatly improve in twenty-four hours the worst of cases.

**Hepatic Disease.**

William Stewart writes to the *British Medical Journal* his experience regarding the use of chloride of ammonium in the hepatic affections of India. The effects are more decided in enlargement dependent on active congestion than on chronic hepatitis and are very remarkable.

**Milk and Quinine.**

If one grain of sulphate of quinine be dissolved in an ounce of milk, we shall find that the bitterness of the draught is hardly perceptible; with two grains there is rather more bitterness, but it is not at all marked; a dose of five grains may be taken in two ounces of milk without any unpleasantly bitter taste, and if the same quantity be put into a tumblerful of milk the bitter is all but lost.

**Intermittent Fever.**

Stern gave carbolic acid in recent cases, as well as in old cases relapsing after quinine. He prescribed it according to the formula of Hehle: Carbolic acid, 0.40; distilled water, 180.00 (or 1 in 72); one tablespoonful three times a day. Out of twenty cases so treated, fourteen were cured after a single dose of this solution, four after two doses; two cases resisted the treatment. Six were quotidian fevers, eleven tertian, and two quartan; in these last there was no return of the attack after the beginning of the carbolic acid treatment; in the tertians there was ordinarily one more attack, in the quotidian two more.—*Canadian Journal of Medical Science.*

**Camphor as a Cure for Tobacco Sickness.**

It is said that a dose of camphor will cure the sickness caused by tobacco. Who has tried it!

**Epilepsy.**

Dr. Hollis, of Brighton, has reported some eleven cases of epilepsy happily treated with bromide of sodium. The dose given ranged from fifteen to forty grains of the salt in an ounce of camphor water or other vehicle three times a day.—*London Medical Examiner.*

**Inflammation of the Bladder.**

Dr. George Johnson has called attention to the happy effects produced in certain cases of cystitis by an exclusively milk diet. The milk is to be taken cold or tepid, and not more than a pint at a time to obviate curdling. Unskimmed milk is the best, as the cream lessens the tendency to constipation.—*London Medical Examiner.*

**Strychnia-Poisoning.**

J. W. Holland, M. D., reports in the *Louisville Med. News* a case of strychnia-poisoning, in which the subject took five grains in four ounces of whiskey; had chronic spasms of muscles generally, loss of volitional power over limbs, and pulse of 120. A mustard emetic emptied the stomach, and he was given bromide of potassium and chloral, was liberally chloroformed, and ultimately recovered.

**Sciatica.**

Dr. Volquardsen reports in Schmidt's *Dictionery* and the *Pesth Medico-Chirurg. Presse,* a case of sciatica which lasted for two years and defied all treatment. He then arrived at the idea of trying the internal use of phosphorus, which he prescribed in doses of fifteen milligrammes (about one-fourth of a grain) three times a day. Three days sufficed to obtain a marked improvement, and three weeks brought a complete cure.—*Lond. Med. News.*

**Nervous Sedative.**

Dr. Lyman refers to the efficacy of nitrite of amyl in a case of the opium habit, where, after two or three nights of complete wakefulness, consequent on breaking off the habit, a nervous irritation had ensued most distressing to the patient and the friends. He had caused the patient to inhale the nitrite directly from the vial; two or three whiffs had been sufficient to bring on the therapeutical effects, which were followed by refreshing sleep. The flushing of the face had been the criterion for ceasing the use of the drug in this case.—*Boston Med. and Surg. Jour.*

**Eczema.**

℞

Acid salicyl, grs. 40.

Tinct. benzoin, dr. ½.

Alcohol.

Glycerinæ, āā q. s. ad solut.

Unguent. resinæ, oz. 1.

M.—Rub in gently twice or thrice a day, after washing with soap and water.

**Pancoast's Styptic.**

Carbonate of potassa, 1 ounce.
Castile soap, 2 drachms.
Alcohol, 4 fluid ounces.

Mix. This styptic has been found preferable to iron solutions in many of the minor cases of hæmorrhage, inasmuch as it leaves the surface of the wound in a healthy condition, and does not produce the thick incrustation so often objectionable after the application of iron.

**Intestinal Obstruction.**

Following the perusal of a paper by Mr. Macrae published in January, 1873, Dr. Norman Kerr, of London, has used belladonna in doses of one and two grains repeated every hour in five cases of intestinal obstruction after other measures had been resorted to without effect. 9, 9, 12, 14, and 16 grains were administered respectively in each case, and the time intervening between the commencement of the treatment and its cathartic effect varied between 7½ and 9 hours.—*Br. Med. Jour.*

**Opium Habit.**

Dr. W. C. Blalock, in the *Atlantic Medical Journal*, recommends hydrocyanic acid as an efficient substitute for opium and morphia, in the treatment of the opium habit. He has found "no patient who could not quit morphia while under the influence of the acid." It acts pleasantly, without depressing after-effects. His formula reads: ℞. Acidi hydrocyanici dil. gtt. xlviij; syrup. simp. ℥ij; acquæ ℥j. M. Sig: a teaspoonful at 7 A. M., 12 M., and 8 P. M.

**Antiseptic Balsam.**

(Dr. J. Felix's "Cicatrizing and Antiseptic Balsam.")—

Acid. Carbol, pur., liquefact, partes 4.
Morphiæ hydrochlorat, partes 1.
Tinct. arnicæ, partes 10.
Tinct. aconiti, partes 10.
Balsami Peruviani, partes 25.
Glycerinæ, partes 50.

M.

Said to be an excellent application to malignant ulcers.

**Vauquelin's Anti-asthmatic Cigarettes.**

Sodium arseniate, 3 grains.
Extract of belladonna, 8 grains.
Extract of stramonium, 8 grains.

Dissolve the arseniate of sodium in a small quantity of water, rub it with the two extracts; then soak up the whole mixture with fine blotting paper, which is dried, and cut into 24 equal parts. Each part is then rolled up in a piece of cigarette-paper.

Four or five inhalations from one cigarette are generally sufficient as a dose.

**Opium Poisoning.**

Dr. William Seldom, of Norfolk, Va., in a communication to the *Virginia Medical Monthly* for October, relates several cases of opium poisoning in which the patients could only be roused by immersing their feet and legs in water, scalding hot. He uses water hot enough to cause severe pain, but tries to stop short of vesication, though in most cases in which he has used it blistering has occurred. He also advocates the plunging of the feet in water scalding hot, or nearly so, in cases of coma or torpor of the brain from various causes.

**Hypodermic Solution of Morphia and Atropia.**

℞

Morphia suph., 24 grains.
Atropia sulph., 1 grain.
Oil bitter almonds, 1 drop.
Water, 2 ounces.

Mix and filter. 10 minims contain ¼ gr. morph. and 1-96 gr. atropia. These amounts have been found by experience to balance each other, so that the full anodyne effect of the morph. is secured without nausea. The oil of bitter almonds prevents all muddiness.

**Epilepy.**

Wharton Sinkler, M. D., has used Canabis Indica in three cases, one of which is given in detail, and is the more valuable. A boy of ten years had epilepsy following a dog-bite, which commenced as *petit mal*, and developed into nocturnal attacks of *grand mal*, occurring about every two hours. Bromide of potassium relieved him somewhat for a time, and oxide of zinc, being given instead, had no curative effect. One-sixth of a grain of Canabis Indica, three times daily, lessened the attacks to daily intervals, for a week, when they entirely ceased, and, at the date of the report, three months had passed without a return of the trouble.—*Med. Times.*

**Hiccough.**

Dr. Favier (*LaFrance Med.*) had treated a patient suffering from hiccough with blisters covered with morphia, moxas to the epigastrium, injections of morphia, ether, bromide of potassium, chloral, etc., without avail, when it occurred to him to compress the patient's epigastrium firmly with the aid of an abdominal tourniquet. In five minutes the hiccough, which had lasted fifteen days, ceased completely. When the compression was suspended, the hiccough at first returned, but shortly disappeared permanently.

**Constipation.**

Dr. L. W. Hanson, of Davison's Station, Michigan, writes: I do not remember seeing in any of the journals or elsewhere any mention made of the use of glycerine as an injection for the relief of a constipated condition of the bowels. For the past two years I have been in the habit of using it in all classes of cases where I desired to produce evacuation of the bowels by enema, whether of acute disease or habitual constipation. I use it, one part of glycerine to six, eight, or ten parts by measure of tepid water, and find it to act more pleasantly and efficiently than any other agent ever used for the purpose.

**Hæmoptysis.**

The sovereign remedy against hæmoptysis is ergotine, says a foreign physician, which, as is well known, excites the vaso-constrictors. A solution in glycerine (1:10) is better than a solution in water, as after long standing it shows but little sediment and no fungi. After the injection the spot injected becomes very sensitive, with some heat, followed by redness, which disappears in eight or ten hours. If the patient is much excited or has much cough, the author is accustomed to precede the ergotine injection with one of morphia, or to give them both at once but in different places. In this way, the patient becoming quiet in mind and body, the ergotine has a better chance to act.

**Chronic Cystitis.**

Charles W. Miles, M. D., of Jordan, Ky., has used the nitrate of silver in solution as an injection in five cases of chronic cystitis, with most satisfactory result. He begins with a solution of two or three grains to the ounce, and increases the strength as the bladder becomes tolerant, the injection being made at intervals of two to three days. He does not resort to the preliminary measure of washing out the bladder [wherein he makes a mistake], but injects the solution after the patient has voided his urine through the catheter [and thereby fails to reach all the folds of mucous membrane]. None of his cases required stronger than twenty-five-grain solutions and most of them yielded to one of ten-grain strength. He uses a silver catheter and metallic syringe; uses a hot hip-bath to relieve severe pain following the injection, or, if it is very acute, he injects olive oil. In connection with this treatment, Dr. Miles employs a mixture containing chlorate, bicarbonate, and iodide of potassium with syrup of buchu, together with mucilaginous drinks.

**Sea Sickness.**

Crochley Clapham, under date of July 29th, says that since the report of cases by him in 1875, he has received many letters from persons who have derived relief from amyl nitrite. Regarding the quantity and mode of use he recommends that it be carried in capsules to prevent evaporation and loss, and says that almost any amount may be inhaled by a healthy person, while under no circumstances would he administer it to a person suffering from arterial disease. A point worth noting in its administration is the necessity for excluding air, excepting so much as may penetrate the saturated portion of a handkerchief from which it should be inhaled.—*Lancet.*

**Glandular Sore Throat.**

Glandular sore throat, by which I mean catarrhal congestion or inflammation in and around the glandulæ of the mucous .membrane of the pharynx and larynx, is a very tedious and troublesome affection. It has been known as dysphonia clericorum; it is, in fact, the chronic sore throat to which persons are liable who use their voices extensively, especially in large rooms or in the open air. I desire to draw attention to the usefulness of the topical application of borax in its treatment. I order a saturated aqueous solution, which the patient applies to his throat by the aid of Corbyn's throat spray. The spray should be employed for several minutes thrice or more frequently daily, and midway between meals. If the larynx be much implicated, the patient should inspire deeply while the spray is playing upon his throat. I have lately found this very simple method of treatment of striking service. The cure may be expedited by the application of astringent solutions to the pharynx and larynx by means of suitable brushes. When there is much secretion, extract of eucalyptus is a good local astringent, which may be used in the form of lozenge.—*James Sawyer, M. D., London.*

**Antidote to Strychnia.**

Dr. Attilio Lelli, having met with a case in which a large dose of strychnia was administered in coffee without fatal consequences, was led to institute some experiments to determine whether it possessed an anti-toxic power against this drug. The animals employed were rabbits, and by comparative trials he found that a dose of five centigrammes proved fatal in a short space of time; when the same or a larger dose was given in a very strong infusion of coffee, he found that the coffee either acted as an complete antidote in preventing the poisonous effects of the strychnia, or that it materially diminished the violence of its action.—*Lancet.*

---

**Whooping-Cough.**

Dr. Z. T. Dellenbaugh, having made use of picrate of ammonium as a remedy for whooping-cough, reports in the *Medical Times* that he has had ten or twelve additional cases, some of which were cured in from twenty-four to seventy-two hours, and he believes the remedy to be a specific for this affection. He gives to babies one-sixteenth to one-twelfth of a grain; to children, one-twelfth to one-eighth of a grain every three hours. He has also used it in one case of diphtheria in the form of a gargle of the strength of eight grains to the pint and by atomization. The picrate causes such a yellowish staining of the parts that he is inclined to think the cure dependent upon a destruction of the micrococci.

---

**Membranous Vaganitis.**

Dr. Parvin says, in the *American Practitioner*, that he had a case of vesico-vaginal fistula, in which, without permanent results, the diseased patches of membrance had been removed, and the surfaces in vain brushed with solutions of nitrate of silver, carbolic acid, and sulphate of zinc, and with tannin in glycerine, frequent cleansings of the parts enjoined, and mucilaginous injections used; but, in spite of all, he says, the membranes appeared again, often within twenty-four hours.

Finally, after the failure of these and various other means, he used large tampons of patent lint smeared with oxide of zinc ointment, and in a week the improvement was so great that he operated upon the fistula. The operation was successful, and after the closure of the fistula there was no return of the diphtheritic deposit.

---

**Poultices.**

The common practice in making poultices by mixing the linseed-meal with hot water, and applying them directly to the skin, is quite wrong, because, if we do not wish to burn the patient, we must wait until a great portion of the heat has been lost. The proper method is to take a flannel bag (the size of the poultice required), to fill this with the linseed poultice as hot as it can possibly be made, and to put between this and the skin a second piece of flannel, so that there shall be at least two thicknesses of flannel between the skin and the poultice itself. Above the poultice should be placed more flannel, or a piece of cotton wool, to prevent it from getting cold. By this method we are able to apply the linseed-meal boiling hot, without burning the patient, and the heat, gradually diffusing through the flannel affords a grateful sense of relief which cannot be obtained by other means. There are few ways in which such marked relief is given to abdominal pain as by the application of a poultice in this manner.—*Dr. T. Lauder Brunton, in Brain.*

---

**Salicylic Acid in Acute Rheumatism.**

DR. S. W. GILLESPIE, of Sterling, Ill., has taken the trouble to collate a number of facts connected with the use of salicylic acid as a remedy for acute rheumatism, which he publishes in the *Chicago Med. Jour. and Exam.* We quote the table which he gives as summary:

| NAME. | Number of patients. | Aver'ge time patient sick in days.. ....... | Ave. time pain was relieved in after treat. commenced.. | Ave. time taken to reduce temp. Hrs. |
|---|---|---|---|---|
| Dr. Sawyer, Eng........ | 3 | 12 | 4 | 6 |
| Dr. Clark, N. Y........... | 5 | 7 | 4 | — |
| Dr. Perrin, Mich.......... | 3 | 12 | 2 | — |
| Leeds Infirmary........... | 9 | — | 2 | — |
| Prof. Traube................. | 14 | — | 3 | 48 |
| Dr. Stricker, Berlin..... | 14 | — | 2 | — |
| Dr. Jacobhouse............ | 45 | — | 3 | 24 |
| M. See, Paris............... | 52 | 13 | 3 | 48 |
| Cook Co. Hospital....... | 18 | 22 | 3 | — |
| St. Joseph's Hospital... | 4 | — | 2 | — |
| Other cases.................. | 33 | 16 | 3 | 24 |
| Total.................... | 200 | Ave, 13.5 days. | Ave. 2.8 days. | Ave. 30 hours. |

NOTE.—In the blank spaces the time was not given.

---

**Dysentery.**

Dr. Washington, of Augusta, Ga., writing to the Nashville *Journal of Medicine and Surgery*, says that, having a very severe case of dysentery to treat, in which there was terrible tenesmus and vomiting, with cold, clammy sweat, and other signs of excessive distress, he gave the patient about one-third of a grain of morphia by puncture, solely with the intent of deadening sensibility; but to his surprise, in a few moments he was perfectly quiet, and the vomiting and purging almost entirely relieved; another puncture completed the work, and the patient was convalescent in a little over forty hours.

After referring to several other cases in which like results were obtained, he adds that he was then attacked himself. There was severe vomiting and tenesmus—in fact, a movement from the bowels every five or ten minutes, and sometimes he could not leave the stool more than three or four steps without having to return. He tried opium, but could not retain it. He then thought of the hypodermic puncture, and although so weak and faint that he could not sit up, prepared the instrument (being alone) and gave himself a good puncture, and, lo! in a few minutes, he was perfectly relieved. He then applied a wet bandage over stomach and bowels, and was soon convalescent.

**The Hair.**

Ammonia should not be used on the hair: it injures the gloss and softness, causing the hair to become harsh and dry. The best way to cleanse the hair and keep the scalp healthy is to beat up a fresh egg and rub it well into the hair, or if more convenient, rub it into the hair without beating. Rub the egg in until a lather is formed; occasionally wet the hands in warm water, softened with borax; by the time a lather is formed the scalp is clean; then rinse the egg all out in a basin of warm water containing a tablespoonful of powdered borax; after that, rinse in one of clear warm water.

**Salt in Milk for Children.**

Dr. Q. C. Smith, of Cloverdale (*Pacific Med. and Surg. Journal*), conveys a valuable hint in the following note: " When cow's milk is found to disagree with hand-fed babies or small children, it may in many cases be rendered entirely wholesome to them by adding to it a small portion of table salt, just enough to be preceptible to the taste. I have for years directed the practice of this expedient among our people, and know it to be of real value."

[Our own experience with milk has been that the addition of salt is always an advantage. The stomach tolerates it better and it is rendered more palatable.]

**Eczema Rubrum.**

L. A. Duhring and A. Van Harlingen send to the *Medical Times* a communication which contains the results obtained from the use of the glycerole in dispensary practice during six months. The formula used was that advised by Mr. Balmanno Squire, of London, and was composed as follows:

Acetate of lead, 5 parts.
Litharge, $3\frac{1}{2}$ parts.
Glycerin, 20 parts by weight.

Mix and expose for some time to a temperature of 350° F. Filter through a hot-water funnel. The clear, viscid fluid which results contains 120 grains of the subacetate of lead to the ounce, and is used as the stock from which preparations of various degrees of strength are afterwards made by diluting it with glycerin. It is most useful in those cases where the affection is extensive, of a dusky hue, accompanied by much weeping, oozing, and infiltration of the skin, together with swelling and œdema of the subcutaneous tissues and a full and varicose condition of the venous circulation. In such cases glycerole of the subacetate of lead, used with diligence, and followed by careful bandaging, constitutes a remedy of the highest value.

**Chronic Alcholism.**

In reply to a question by a correspondent in the *British Medical Journal*, regarding the best treatment for the tremors of chronic alcoholism, and a substitute for the constant craving for drink which exists, Dr. Lauder Brunton recommends fifteen minims of tincture of perchloride of iron, with ten minims of tincture of nux vomica, as most efficacious for the tremors, combined with bromide of potassium, if restless at night. The chalybeate mixture, either alone or with the addition of tincture of capsicum (five or ten minims), relieves the craving for drink, for which purpose also a mixture of carbonate of ammonia in infusion of gentian is valuable. If there be derangement of the stomach, it should be treated by ten-grain doses of subnitrate or carbonate of bismuth, with magnesia and tragacanth.—*London Med. Record.*

**Cholera.**

Surgeon W. J. Butler, of the Madras Medical Service, calls the attention of the medical profession, through the *Lancet*, to the value of boracic acid in the treatment of cholera. He states that having had considerable experience in the treatment of the fatal malady in the course of numerous and extensive epidemics in Burmah and Southern India, and having employed all the various treatments which have had any claim to success, with very poor results, he was induced to consider whether any more efficacious remedy could not be resorted to. At the period when the properties of boracic acid were made public he determined to try its effects.

The pure acid not being procurable, the biborate of soda (borax) was at first used, and with marked benefit, the percentage of recoveries being from 70 to 75 per cent. Subsequently he has used the pure acid in ten grain doses every two hours, combined with borax or bicarbonate of soda, under which treatment every case has recovered. He adds that in no case were any signs of irritation or ill effects observed from the remedy; and that in all of them the renal secretion was re-established with much greater facility than under any other method of treatment.

**Tape-Worm.**

Mr. C. H. Cressler has communicated to the *American Journal of Pharmacy* the result of some experiments made by him to test the efficacy of the marginal shield-fern (*Aspidium marginale*) in the expulsion of the tape-worm. He was led to make these trials through the failure, on a certain occasion, of an emulsion of the oleoresin of the imported male fern (*A. filix mas.*), to effect satisfactory results. As might have been expected from species so nearly allied, he found the constituents of the latter to be identical with those of the male fern, and its efficiency to be fully equal. The marginal shield-fern is one of our most widely-distributed species of the North, is very common in all of our woods, and, being an evergreen, may be collected at any season of the year. Most of the male fern for medicinal use has hitherto been imported, although the plant is found northward in the region of Lake Superior, and westward. Mr. Cressler's experiments were made with the oleoresin obtained from the rhizome of the fresh plant, and administered in gelatine capsules.

**Typhoid Fever.**

Dr. P. L. O'Neile, physician to the Fever Hospital, Athy, says, in the *Practitioner:*

In the April number of the *Practitioner* there appeared an extract from a communication of Dr. Persse White to the *Medical Times and Gazette*, in which he extols oil of turpentine as a remedy in bronchitis complicating typhoid fever. I can endorse his remarks, and at the same time add that oil of turpentine, combined with sweet oil, in the proportion of fifteen minims of the former to thirty or more of the latter for a dose, and made up with mucilage or the yolk of an egg, is the best remedy for the diarrhœa and tympanites, which are generally associated with the typhoid which prevails here at present. In the extensive practice which I enjoy, I have found this combination superior to any other drugs. Under its influence the stools are reduced in number, and become more solid in consistence, and the tympanites gradually less. In only a few patients have I known its administration to be attended with disagreeable results to the stomach, the greater number manifesting a wonderful tolerance of it. It may be given three or four times in twenty-four hours, and continued for weeks, if necessary. It prevents the palpable wasting of the disease, and obviates the necessity of giving alcoholic stimulants to a great extent—no mean desideratum in a workhouse institution.

**Cardiac Affections.**

Dr. Angrisani, in an interesting article on this subject, gives the following conclusions:

1. Bromate of potash has a depressing action on the vaso-motor centres and on the cardiac plexus.

2. This effect is produced in the vaso-motor centres by a mode of action which is quite peculiar to it, and which we do not understand, and not because the bromate acts on the smooth fibres of the capillaries. The diminution of the lumina of the capilliaries may depend simply on the extension of the action which this salt exerts on the centres and the vaso-motor nerves alone in physiological experiments.

3. The bromate has no action on the muscular fibres of the heart like digitalis, and the latter has no action on the arteries.

4. The bromate is the most suitable medicine for correcting the functional anomalies of the heart, such as frequency, intermittence, arhythm, etc., whatever may be the condition of the miocardium.

5. It modifies rapidly and advantageously angina pectoris and palpitations when they are simply neuroses. In those cases which depend on profound anatomic pathological changes of the heart and vessels, or on compression, the bromate is still capable of producing an amelioration which is more or less durable.—*From Lo Sperimentale in N. Y, Jour. Medicine.*

**Asthma.**

The Paris correspondent of the *British Medical Journal* writes: For nearly twenty years back, M. See has been employing the iodide of potassium for the cure of asthma, and, according to his experience, it may be looked on as a specific for the disease, if there be such a thing as a specific in medicine. It is true that others had employed it, and are still employing it, in this affection; but, as it is invariably prescribed with other substances, such as ipecacuanha, opium, belladonna, ether, etc., it is difficult to say to which to attribute the curative effect. M. See has had the idea of trying the iodide of potassium alone, which has been followed with the happiest results. He prescribes it not only during the attack, but enjoins the patient to continue it for weeks, months or years, according to the severity or duration of the malady. In exceptional cases he combines it with a little opium, to prevent iodism, and, when the breathing is greatly compressed, with chloral. During the paroxysm, however, M. See employs the iodide of ethyl, a substance discovered in 1825 by Gay-Lussac, and composed of iodine and ether, the new compound possessing the respective properties of both these substances. He administers it by inhalation, and he has often found that a single dose of five or six drops has been sufficient to cut short a paroxysm. The breathing once relieved, he then trusts to the iodide of potassium to effect a cure. The above treatment has been found useful in all cases of asthma, whatever its origin; and the iodide of ethyl has also proved efficacious in relieving cardiac and laryngeal dyspnœa.—*Med. and Surg. Rep.*

---

**Dressing for Wounds.**

Dr. T. G. Nasmyth, M. B., C. M., Edinburgh *Medical Journal* for March, gives this note as to the use of terebene:

The first case that I saw treated by this was a gunshot wound of the hand, where several fingers were blown off and the hand much lacerated, and, from the nature of the injury, irregular and ragged flaps were left where the destroyed fingers had been removed. Terebene, in the proportion of one part to six of olive oil, was used from the first, there never was any putrefactive odor from the wound, very little sloughing took place, and the wound granulated kindly without any bad symptoms occurring. Its use was tested in another case, one of excision of hip-joint, where the acetabulum was diseased as well as the head of the femur. Terebene was applied on lint to the surface of the wound, and during the first day or two after the operation into the cavity of the wound; in this case there never was any bad odor either, and the large wound filled up very rapidly. Terebene seems to stimulate granulations more than carbolic applications. A wound of the scalp in a drunkard, who fell from a two-story house, healed without the slightest bad effect occurring, and in several other scalp wounds a like result followed. A case of gangrene, occurring in an amputation below the knee, and delirium tremens also complicating the case, tried terebene as a deodorizer thoroughly, and it answered satisfactorily. The smell from the gangrenous stump was kept down, the gangrene did not extend, and very little sloughing occurred. The patient recovered. As an application to ulcers where there is a great amount of discharge, it is a good application, it diminishes the amount of discharge, and prevents the bad odor even in the most fetid ulcer. As an application to burns, it acts just as well as carron or carbolic oil, and it certainly is a more agreeable substance to use than the former. Although the number of cases in which I have seen it used is not very large, still they were cases which tried its value thoroughly, and I have found it very useful. In cases where antiseptics cannot be found—and these do occur especially in the country—terebene will be found very useful.

---

**Epilepsy.**

Dr. Crichton Brown has contributed to the last volumes of "West Riding Lunatic Asylum Medical Reports," a paper in which he describes his experience in the use of nitrite of amyl in epilepsy. The results at which he has arrived are mentioned in the *Medical Press and Circular:*

Being engaged in tracing out the areas of blushing as induced by nitrite of amyl in different individuals and under different circumstances, with a view to elucidate the laws regulating the diffusion of that form of emotional expression, Dr. Crichton Browne was struck by the fact that the degree and extent to which the blushing caused by nitrite of amyl is manifested are influenced by certain pathological states. He found that general paralytic patients may inhale a considerable amount without displaying any marked flushing, even of the face, and that epileptics cannot breathe the smallest quantity without exhibiting extreme cutaneous hyperæmia over the face, neck, and chest. Guided by these observations and by an ingenious argument founded upon them, he was led to conclude that if the nitrite of amyl could be given immediately before an epilleptic fit, the spasm of vessels might be prevented, and so the whole sequence of morbid events averted. "And," as he forcibly remarks, "a fit averted in epi lepsy is no slight gain; it is in fact, a step made toward recovery, and a postponement of those degenerative consequences which are, as a rule, developed in proportion to the frequency and severity of the fits. To interrupt a pathological habit is to give a chance of recovery; to control the fits is to limit the destructiveness of epilepsy." In several cases in which the nitrite of amyl was administered immediately after an aura the usual fit did not supervene, and in one case in which it was administered regularly three times a day, a series of fits from which the patient was suffering was abruptly interrupted. In rabbits, too, rendered artificially epileptic by Professor Ferrier, it was noted that the fit which invariably followed an electrical irritation applied to the exposed brain when no interference took place, was arrested by the inhalation of nitrite of amyl. "The result of all my experiments is to convince me," says Dr. Crichton Browne, "that it will be found invaluable in many cases in not only postponing but in altogether preventing epileptic seizures. The utility of an agent possessing this power can scarcely be exaggerated. It will, I believe, supersede other methods of attempting to avert the fit by acting upon indications afforded by the aura."

---

**Dysentery.**

Dr. Wm. L. Newell, in Philadelphia *Medical Times:* A weak solution of chloral on chronic ulcers manifested such favorable results in my hands that I conceived the idea of using it locally on the inflamed and congested bowel in dysentery. The first case had been under the usual treatment for three days without relief. The child, aged eleven, was tormented with thirst, pain, and tenesmus, with twenty-five or thirty dejections in twenty-four hours. In connection with other treatment I ordered five grains of chloral hyd., dissolved in two ounces starch gruel, thrown up the bowel with considerable force from a hard rubber syringe. It remained three hours, during which the child slept. Many of the other symptoms were modified, and the injection was repeated, which remained seven hours, when it came away with some fæcal matter, but without tenesmus. The child asked for food, which was given in the form of mutton tea thickened with boiled wheat flour. All treatment ceased in forty-eight hours from first enema, four being given in all. The case seemed so satisfactory that I mentioned it to my *confrere*, Dr. J. S. Whitaker, who has pursued the same treatment with the most happy results in every case, aborting the disease within a few hours. I may mention that he used ten grains instead of five with a lady, aged twenty-five years, who had twenty or thirty calls in twenty-four hours, with complete repose for eight consecutive hours, with permanent abatement of all other symptoms, without other treatment.

**Rheumatism.**

In a clinical lecture reported in the *Medical and Surgical Reporter*, Prof. Da Costa says:

"The treatment of rheumatism by salicylic acid and its salts is comparatively a new one. I will not say that I have entirely and fully accepted this new treatment. I claim for it, however, that where it acts it does so with great rapidity. Summing up my present belief, I would say that where you have a case which is on saliclylic acid, and does not do well in three or four days, abandon the treatment. It acts by lessening the pain and reducing the temperature, therefore, on the fever processes as well as on the rheumatic poison. It certainly gives marked relief from the articular pains, but I have noticed in some cases a decided depression following full doses.

"I have endeavored to obtain a method of giving salicylic acid in solution, which would be pleasant and not disagree with the stomach. I now use an agreeable combination which, at the same time, holds it in solution. I will not stop to discuss the pharmaceutical questions in regard to the chemical considerations involved, but will merely state that the form that I have found to best answer this purpose is salicylate of soda, where we have the acid in a soluble form, agreeably administered in the following formula:

℞

Sodii salicylatis, gr. xx.

Spiritus lavandulæ, ℳ xv.

Glycerinæ, f ʒ j.

Aquæ, ℥ ss.

"This prescription is to be given every three hours until amelioration occurs."

---

**Cleansing the Bladder.**

Dr. Berthole, (*Cbl. f. Chir.*, from *Gaz. Hebdom,*) suggests a method of injecting fluids into the bladder without the use of the catheter, a plan which, although not new, has not receieved the attention which perhaps it deserves.

It is known that if a stream of water or other fluid is introduced into the urethra it will, if entering under sufficient pressure, gradually dilate the sphincter vesicæ, and it may be caused to enter the bladder when through inflammation or otherwise the urethra is so sensitive as to prevent the passage of a metal or gum catheter.

In using the injection by Berthole's method the patient sits on the floor with his back against the wall, thighs and knees turned out, and the toes turned in. A vessel is placed conveniently to catch any water which may escape. An irrigator with a long tube, with a stop-cock somewhere in its course, is placed upon a bench near by. The tube of the irrigator terminates in a cannula of hard rubber twelve to fourteen centimetres long and six millimetres in diameter, which is well oiled and inserted into the urethra, and the patient keeping this in place with the left hand, can easily regulate the flow of the fluid with his right hand upon the stop-cock. When the latter is opened, the water usually penetrates into the bladder without the patient's being conscious of its entrance. So soon as he feels the desire to urinate, the stop-cock is to be turned off, as the bladder is then full. The patient can now empty the bladder at once or can retain the fluid some little time. The water should be warmed to the temperature of the body, and the best time for employing the injection is just before going to bed. A single injection, in cystitis, will thin the stagnant urine and deprive it of its irritating quality.

The indications for the direct injection of water (or medicated fluids) are as follows: 1. Diseases of the bladder, and particularly chronic essential or consecutive cystitis. In the former, B. considers this method specific; in consecutive cystitis it is only palliative, of course, since the cause (stone, etc.) must be removed. 2. Contraction of the neck of the bladder. 3. Diseases of the prostate, the injection here being directed against the consecutive vesical catarrh. 4. Diseases of the urethra—in particular, urethritis of the deeper portion, where contraction of the membranous portion and of the sphincter vesicæ, with consecutive catarrh, is present. Contra-indications: 1. Paralysis of the bladder. 2. Hypertrophy or other diseased condition of the prostate interfering with urination. 3. Stricture.—*Phila. Med. Times.*

---

**Alcoholism.**

The Superintendent of the New York State Asylum for Inebriates, furnishes some of the prescriptions which have been found useful in the treatment of the patients:

℞

Acidi phosphoric, dil., ♏ 10.

Elixir calisayæ, oz. ½.

M.—Sig.—To be taken at one dose, and repeated before each meal.

℞
Tincturæ nucis vomicæ, gtt. 10.
Tincturæ cinchonæ co., oz. 2.

M.—Sig.—To be taken in water before meals.

℞
Spt. ætheris sulph. co., oz 1.
Tincturæ nucis vomicæ, gtt. 10.

M—Sig.—To be taken when the patient suffers from great restlessness

℞
Extract hyoscyami fld., ♏ 20.
Chloral hydrat, grs. 20.
Aquæ, *q. s.*

M—Sig.—For insomnia.

℞
Extract fluid hyoscyami, ♏ 30.
Sig.—In insomnia, and repeat if necessary.

℞
Extract fluid conii, ♏ 30.
Potassi. bromidi, grs. 20.
Aquæ, *q. s.*

M—Sig.—Repeat in cases of insomnia.

℞
Potass. bromidi.
Sodii bromidi, āā grs. 20.
Aquæ, *q. s.*

Sig.—To be repeated, if necessary, in cases of insomnia; particularly useful where there is marked restlessness.

For the tremor of alcoholism, Mr. Lauder-Brunton has found a mixture of iron and nux vomica the most useful remedy. He gives fifteen drops of the tincture of nux vomica and twenty drops of tincture of iron at a dose. When the stomach is deranged, he administers bismuth and magnesia in equal parts, for some time before having recourse to the iron. When there is a tendency to insomnia he gives bromide of potassium at bedtime. When the desire for alcoholic drinks is great, he adds ten to fifteen drops of the tincture of capsicum to the mixture of iron and nux vomica. If this fail to have the desired effect he often uses a mixture of magnesia, sulphate of iron and oil of cloves, or an infusion of gentian with carbonate of ammonia. When the desire for alcohol returns at more or less distant intervals, it may be controlled by the bromides.—*British Medical Journal.*

---

**Elastic Pencil of Lunar Caustic,**

Is prepared by Dr. Pajot by dipping a laminaria tent two millimeters in diameter into tolerably thick mucilage and then rolling it in finely powdered nitrate of silver. After drying, an elastic pencil the size of the ordinary stick remains which may be readily introduced into the uterine or other cavity without fear of fracture.

---

**Cement for Sealing Bottles, etc.**

Mix 3 parts of resin, 1 part of caustic soda, and 5 parts of water. This is then to be mixed with half its weight of plaster of Paris. The compound sets in three-quarters of an hour, adheres strongly, is not permeable like plaster alone, and is affected but slightly by warm water.

